# ‘All together now’: Facilitators and barriers to engagement in mutual aid during the first UK COVID-19 lockdown

**DOI:** 10.1371/journal.pone.0283080

**Published:** 2023-04-12

**Authors:** Chris Cocking, Sara Vestergren, Evangelos Ntontis, Katarzyna Luzynska

**Affiliations:** 1 School of Humanities and Social Sciences, University of Brighton, Brighton, United Kingdom; 2 School of Psychology, Keele University, Newcastle, United Kingdom; 3 School of Psychology and Counselling, The Open University, London, United Kingdom; University of Nairobi, KENYA

## Abstract

Despite undeniable hardship, the onset of the COVID-19 pandemic also saw an outpour of community solidarity and mutual aid towards those in need. This study explored why people participated in mutual aid during the pandemic as well as the factors that contributed to continued involvement and/or its decline. We conducted remote interviews with 17 people in South-east England who had been involved in volunteering and local community mutual aid support groups during the first UK lockdown from March to May 2020. Using thematic analysis, we identified two themes: 1) *The emergence of social groups and their psychosocial effects*, and 2) *Enduring connections and barriers to continued participation*. Participants often reported an emergent shared identity, preferring the localised nature of these groups and the associated mutual nature of support. They also reported intentions to continue providing such support, should the need arise again, and any barriers to continued involvement in mutual aid were better explained by structural and systemic issues, rather than individual motivational factors.

## Introduction

The COVID-19 pandemic has brought immense suffering and loss since it first emerged in late 2019, with national lockdowns and other protective measures imposed to prevent the spread of infection. Such measures acutely affected individuals and communities on a global scale and remained for prolonged periods in many countries. For instance, the emergence of the Omicron variant in late 2021 forced the UK government to impose ‘plan B’ restrictions, such as working from home where possible and encouraging the use of face masks [1], and in April 2022 there were nearly 5 million recorded cases in the UK (or 1 in 13 of the population) [2]. However, as restrictions were first imposed, we also witnessed mass outpourings of solidarity, rapid increases in volunteering, and the provision of social support [3–5]. These cooperative activities among others included: shopping for vulnerable people and/or those self-isolating; collecting medical prescriptions; and providing emotional support and/or information. Between March and May 2020 over 4,300 mutual aid groups were created, comprising approximately three million people in the UK and over 750,000 people responded to a call for volunteers to help the National Health Service (NHS), although a significant proportion of those volunteering were not utilised [6].

Such cooperative behaviour is not uncommon in the face of adversity, as extreme events are often characterized by an emerging sense of camaraderie and mobilization of social support [7]. Social psychologists have argued that the experience of common fate and shared adversity (i.e., being affected by the same event) can help people share an emergent social identity and perceive each other as members of the same social group (e.g., ‘*us* the disaster survivors’). This shared social identity can operate as the psychosocial basis for the mobilization and provision of social support and solidarity. This process is encapsulated within the Social Identity Model of Psychosocial Collective Resilience (SIMPCR) [8, 9]. However, the shared social identity that emerges during the early stages of disasters (and by extension the various cognitive, emotional, and relational changes associated with its emergence as well as its potential benefits) can dissipate over time [10]. For instance, community identity can decline because of a lack of perceived common fate, due to inequitable treatment by the authorities, or disidentification from a disaster survivor identity [11]. Concerns were also expressed in a worldwide study about possible ‘pandemic policy fatigue’ (p.1) with relation to reductions in some protective behaviours among the public [12]. However, the decline of adherence to the behavioural restrictions imposed in the UK during the COVID-19 pandemic was more likely caused due to systemic failures and lack of trust in government [13]. In this paper we extend the previous literature in two ways. First, we examine people’s motivations to engage in volunteering and mutual aid during the first national lockdown in early to mid-2020 in the UK. Second, we explore why such participation endured or what were the barriers to sustained engagement.

### Volunteering and social support in emergencies

Despite evidence regarding the endurance of shared social identity in disasters, less is known about the ongoing influence of such processes in the context of disaster volunteering. Recent research has found that social networks and connections, local knowledge, and social trust were associated with engagement in mutual and social support when volunteering during the COVID-19 pandemic [14]. The social support that arises from volunteering during emergencies is crucial for encouraging effective community resilience and is recognized in official UK emergency planning guidance [15]. Among other factors (e.g., economic resources), community resilience can also be facilitated through social networks such as the following: a) geographical communities (people from the same area); b) communities of interest (such as sports clubs); c) communities of identity (such as religious, gender, etc) and d) communities of circumstance (people brought together by the same incident). The importance of strong networks for community resilience in extreme events has been theorized through the concept of *social capital*, which emphasizes norms of reciprocity, mobilization of social support, reduced opportunism, and facilitation of social interaction and collective action, highlighting how cohesive communities can respond positively to extreme events [16]. Such cohesive social groups can be crucial for facilitating preparedness before a disaster and are often held together by participants having a shared social identity [17].

As well as through pre-existing groups, social support can also be mobilized in emergencies via communities that operate autonomously of pre-existing networks and provide support directly to those in need [7]. This is often described as mutual aid and such support can go beyond the paternalistic relationship between helper and helped that is common in traditional charity work [5]. Mutual aid focuses instead on more horizontal and reciprocal interactions and is often described as ‘solidarity not charity’ (p.415) [18], with such mutual aid apparent during the early stages of the COVID-19 pandemic [19, 20]. Spontaneous communities providing mutual aid can emerge through the shared experience of a common fate caused by the widespread and generalised impact of major incidents [10]. Furthermore, the spontaneous mobilization of solidarity during the COVID-19 pandemic can be better explained by social identity models of emergency behaviour (as opposed to more individualistic and/or evolutionary perspectives [21]), whereby adverse experiences can result in the emergence of a shared social identity that may not have existed prior to the pandemic [8, 22, 23]. Any variations in this shared social identity are best explained by the strength (or weakness) of a sense of common fate among those affected by the emergency [9] and can also arise when people perceive themselves as being victims of similar systemic inequalities [24]. Shared social identity can in turn mobilize the provision of social support as well as improve people’s wellbeing and sense of collective efficacy, thus also enhancing community resilience [25, 26]. Similarly, shared identity can also encourage more cooperative social and/or behavioural norms, such as: expectations of support from fellow ingroup members, mobilization of social support to ingroup members, and collective coordination in response to a shared threat [8, 25]. The importance of emergent groups and the social support they mobilize, both during the response, as well as in the recovery phase of disasters, has been empirically examined across a range of extreme events such as terrorist attacks [27, 28] and flooding [24, 26]. Furthermore, some of the cooperation towards others during the acute phases of extreme events (such as helping strangers during a terrorist attack) cannot simply be explained by any individual or genetic propensity towards altruism, as there can be a clear and present danger to one’s own personal safety in these situations which could take precedence over any innate cooperative tendencies towards others [28].

Evidence from the COVID-19 pandemic shows that cooperation has been mobilized by both pre-existing and emergent groups. Previous experience, collaboration between volunteers, community organizations and public agencies, have all played a role in addressing needs arising during the pandemic. The role of volunteers in China when COVID-19 first emerged was particularly important since they were familiar with local community norms and had good relationships with other residents [29]. The support provided by volunteer groups can help vulnerable people adhere to lockdown measures through providing essential supplies, medicine and well-being checks [30]. Thus, community level organizations can play key public health roles in pandemics, through facilitating support during both the response and recovery stages [31].

The literature on co-operative behaviour in emergencies points to a range of factors that are associated with the mobilization of prosocial behaviours. For instance, provision of support is associated with increased levels of perceived social support, and a stronger sense of community identification after severe flooding [26]. More recent work has found that prosocial behaviour can lead to significant increases in donations to emergency funds, intentions to learn about COVID-19 related volunteering, and increased trust [31]. Being involved in helping others can influence volunteers’ sense of community belonging, support, and well-being, thus contributing to a ‘social cure’ in the community p.1523 [32]. Having a shared identity with others helping promotes affiliation, meaning and social support (which is often the psychological basis for group behaviour [33]), and can also facilitate the mobilization of social support and increase individual coping [34]. While individual motivations for co-operation are undoubtedly important, empathy has been found to be a stronger predictor of providing social support when the recipient was perceived to be an ingroup member of a relevant social group [35], and volunteering behaviours can be influenced by personal identification and/or a shared sense of national identity with the victims [36]. Similar findings come from the context of COVID-19, with pre-existing identification as a volunteer predicting later helping during the pandemic [37], community and national identification positively predicting provision of social support [38] and reported mental health benefits for those that helped others during the pandemic [39]. Furthermore, in an interview study of community organisers of mutual aid groups, group process strategies and positive emotions were linked to sustained involvement, enhanced feelings of efficacy and greater community cohesion [40]. Finally, in an overview of the potential role that social and behavioural sciences can play in COVID-19 response, it has been suggested that local cooperation can be explained from an evolutionary perspective that sees the concept of personal self-interest extending to looking after family members to increase genetic fitness, and that this may explain why people often prioritise familial over global and/or international interests [41] (although this may be less relevant if there are no family members in one’s own neighbourhood).

### The present study

Based on the existing literature, there are two main gaps that need further exploration to better understand how to facilitate cooperative behaviour in pandemics and other extreme events. Firstly, most previous research on co-operation in mass emergencies did not focus on pandemics prior to the onset of COVID-19, and the studies that have since, have either been aimed at organisers (as opposed to grass-roots activists) [40] or have focused on subjective well-being arising from involvement [42]. Hence, there is a need to explore further the influence processes of social groups and more generally, how a shared sense of identity emerges in pandemics, and what the motivations for such engagement are. Second, while previous literature has focused on predictors of helping [32, 34, 37], evidence regarding the endurance or decline of social support activities remains limited.

Therefore, this study will explore volunteering and mutual aid during the first national UK lockdown from March-May 2020 when communities were faced with unprecedented peacetime restrictions on their daily activities. For instance, by mid-March 2020, UK COVID-19 infection rates and hospitalisations were rising exponentially, and there were fears that the NHS could be overwhelmed with COVID-19 patients. This resulted in a national lockdown instructing people to stay at home except for essential activities that was maintained until restrictions began being eased from 13/5/2020. The lockdown restrictions were especially acute for those that were vulnerable to COVID-19 infection and severe illness and/or death and so may not have been able to leave the house at all during this period. People in such vulnerable groups would have been in more urgent need of support from their local community in the possible absence of support from existing national structures. Therefore, we need greater understanding of co-operative behaviour during this period of the COVID-19 pandemic, and more specifically, how to facilitate community cooperation throughout such ongoing incidents, as well as what potential barriers to such cooperation there might be.

The following specific research questions will be explored:

1. What were participants’ motivations for involvement in helping others during the first UK lockdown and what were their perceptions of such involvement?
2. What are the factors associated with enduring participation in helping others, and what are the potential barriers to sustained engagement?

## Method

### Design and participants

Qualitative research methods were used in this study because of their ability to explore in detail the variety and complexity of participants’ experiences and views of their involvement in helping others during the pandemic. Therefore, we conducted individual interviews (using purposive sampling) to capture the richness of participants’ motivations and experiences. Interviews were semi-structured to enable us to explore our own research questions in depth, but also to allow for possible flexibility and/or novelty within participants’ accounts.

Recruitment adverts were circulated via the lead author’s University webpages, all authors’ social media accounts and their personal contacts, resulting in 17 participants (3 males, 14 females) being interviewed. All participants were involved in local volunteering and/or mutual aid groups during the first wave of the COVID-19 pandemic, covering the period of the first UK wide lockdown between March–May 2020. Participants’ demographic details are outlined in Table 1 below and can be summarised as follows: ages ranged between 32 and 76 (M = 52.72, SD = 12.26); eleven were working and seven retired or unemployed. Six participants were already involved in volunteering for existing support networks pre-pandemic; seven became involved in new mutual aid networks that emerged during the pandemic; and four were involved in both. Participants’ occupations were mainly from public sector and/or health/social care backgrounds, suggesting an existing cooperative professional identity within this sample. Ethical approval for the study was obtained from the lead author’s local Research Ethics Committee. Following restrictions on face-to-face data collection (to prevent risk of COVID-19 infection), audio interviews were conducted remotely (via MS Teams and Zoom). All four authors were involved in interviewing participants. Interviews were conducted in July-August 2020 when strict national lockdown restrictions had been eased.

**Table 1:** Participant demographics outlining age and occupation status.

Pseudonym (Gender)	Age	Occupation status
Ashley (F)	40	Part-Time
Charlie (F)	57	Retired
Sydney (F)	46	Part-Time
Frankie (F)	59	Self-employed
Jules (F)	66	Retired
Alex (F)	65	Retired
Max (F)	32	Unemployed
Sandy (F)	76	Retired
Morgan (F)	54	Self-employed
Jesse (F)	48	Self-employed
Daryl (F)	49	FT
Taylor (M)	32	FT
Stevie (F)	42	FT
Hayden (F)	62	Retired
Corey (M)	68	Retired
Toni (M)	58	Part-Time
Rudy (F)	46	Full-Time

After the interviews, participants were de-briefed and informed about the study’s objectives and offered a £5 voucher in recompense for their time (most declined the voucher and/or asked for it to be donated to a charity of their choice). Interviews lasted between 15–42 minutes (M = 26.24, SD = 8.74, total duration = 472.3 minutes), using a mixture of open and closed questions to explore motivations for involvement, participants’ views of their involvement, and thoughts of others involved (see S1 File for the interview schedule). Pseudonyms are used throughout the manuscript, and any data that could lead to identification of individuals, locations, or organisations, was removed.

### Analytic procedure

Interviews were transcribed verbatim by an approved transcribing service and were checked for accuracy by the interviewers (all four authors were involved in data analysis). We conducted a thematic analysis [43] which was informed by the social identity approach in social psychology and particularly by its application in emergencies. We conducted multiple readings of the transcripts, taking notes related to the study’s aims. As we aimed to explore factors relating to engagement in mutual aid groups, we applied selective coding to our data (e.g., by creating codes for relevant extracts of the transcripts such as: enhanced/emergent shared identity; reasons for involvement; perceived efficacy of actions; enduring commitment and/or potential barriers to involvement etc). The codes were then analysed for similarities in terms of meaning and subsequently organized into two distinct and coherent overall themes, which are presented below and are supported by quotes from the dataset.

## Findings

From our analysis of the interviews, we created two broad themes relating to participants’ views of their involvement in helping others during lockdown as follows: 1) *The emergence of social groups and their psychosocial effects* and 2) *Enduring connections and barriers to continued participation*. Both themes will be addressed in turn.

### The emergence of social groups and their psychosocial effects

This theme explores participants’ sense of shared identity with others, and whether these connections existed before the pandemic or if they emerged from their own experiences of helping others. The theme is organised under two sub-themes. In the first sub-theme *(opportunities to create a group*), we explore the extent to which participants felt that the common experience of the pandemic had facilitated a shared identity, and how a sense of connection emerged through participants’ involvement in helping others. In the second sub-theme *(shared social identity and social support)* we discuss how participants’ involvement provided them with a sense of mutual support that resulted in more inclusive shared social identities that transcended any previous group boundaries between helpers and helped.

#### Opportunities to create a group

A sense of shared identity was generally considered important by participants and was also used to explain their own involvement in helping others. However, this expression of shared identity was more nuanced at times, and while some participants reported an existing sense of shared identity that pre-dated the pandemic, this was not experienced by everyone. For example, Taylor initially reported a low sense of shared identity with their local community:

*Int*: *Did you feel part of the local community before the pandemic?*

*Taylor*: *No*, *is the short answer… my immediate neighbours either side*, *I have a good neighbourly relationship with them*, *and I think two other neighbours further along*. *I wouldn’t call that feeling like part of the community*. (Taylor)

Toni reported that their involvement enabled an increased sense of connection that helped them overcome their previous difficulties in achieving such integration within the town that they had recently moved to:

*I think it’s sometimes quite difficult to get a sense of community in Brighton…I’ve heard this said to me*, *by people who move into the town*, *that it’s actually a friendly town*, *but it’s not particularly sociable in that it’s…quite difficult to get into circles…that’s been my experience as well*…*Brighton is a community and there’s loads of great community stuff going on*. *I guess I’m saying that I’ve never really found a way of tapping into it until fairly recently*. (Toni)

So, even though Toni was living in the area before the pandemic, they had struggled to find a way to become part of the community. However, the response to the pandemic offered new opportunities to get involved. For some, the increased sense of community cohesion from supporting others developed quite quickly during the first lockdown in March 2020. Max also emphasised the opportunity that the pandemic created to become part of the community. They were new to the local area and reported developing a higher sense of community with the people living in their street than they had previously felt before the pandemic began. In a sense, the isolation through lockdown resulted in emergent social relations via new pathways that may not have been apparent previously.

Participants also reported that since becoming involved in their local mutual aid group, they had developed new personal connections with most of the people in their street, thus supporting the notion that new social bonds had emerged from their involvement:

*[I] moved into where I’m living now just before lockdown*…*I didn’t actually know people*, *particularly*, *on our street*…*but now*, *pretty much*, *know everybody by name*. *That’s the level of community that I don’t think I had before* (Max)

However, this sense of enhanced common identity that emerged from shared experiences of the pandemic was not limited to newcomers and some participants who already had an established connection with their local community also reported increased connections resulting from their involvement in helping others. For example, Stevie who had lived in their local area since childhood, became involved along with other family members in supporting others during the first lockdown. Consequently, having provided such support for others, they stated that they now felt closer to the local community above and beyond any pre-existing sense of connection that they had experienced previously:

*I think in terms of how we feel about our community and our role in the community and the idea that you do look out for one another and you do take care of each other when you are in a community*…*I think that has definitely been enhanced*. (Stevie)

Here, Stevie also highlights the reciprocal nature of being part of a shared community, the mutual care, and general sense of support. The participants in our dataset expressed feelings of care and protectiveness for their fellow volunteers, even in the absence of face-to-face contact. This suggests that this new, emergent sense of shared identity extended beyond the traditional social relationships involved in the creation of friendship bonds in pre-pandemic times, such as physical proximity to others and personal contact (see [44] for further exploration of possible ways of maintaining social connections during the COVID-19 lockdowns). Thus, the creation of virtual social groups was important in creating strong social bonds that generated a sense of protectiveness to other group members, despite never having met them, as Charlie illustrated:

*Some of the other ones I’ve never met face to face*. *But I feel very protective of them* (Charlie)

Some social support networks were also set up via social media with the explicit intention of reaching those who might not be able to access support via other social media platforms, as illustrated by Taylor, who set up an online group to try and reach those in their street:

*The reason for me setting up the WhatsApp group was that I know lots of people won’t be on Facebook or won’t know about these things*. *It did seem like the physical proximity to the people around me was the thing that needed support*. *I felt more like people who were on the Facebook mutual aid pages*, *they are*, *by definition*, *connected to some support network*. *I had no idea whether my next-door neighbours did have that kind of thing* (Taylor)

There was also a recognition that the support given could go beyond provision of tangible goods and/or services and could also be effective in generating a sense of social support on top of any specific instrumental support given. For instance, Taylor describes how some responded to their offers to help by saying that they preferred seeking social connection rather than any personal need for practical help. Consequently, Taylor reported that their increased sense of community (which they had previously felt was low) came about from others’ appreciation of them producing leaflets offering support:

*Three people telephoned me…the day after that I delivered these leaflets saying that they were fine but that it was good to have had the note put through the door*. *Of those*, *two of them said that they were interested in companionship…This is how I came to be more connected to my community…People were ringing me up and saying*, *“I would like to help*,*” or*, *“Thank you very much for the flyers*. *We don’t need help*, *but it is really nice to know that there is somebody there to call* (Taylor)

#### Shared social identity and social support

Participants’ sense of increased connection with their local community was often explained by a general feeling that they were contributing towards a mutual collective effort and a belief that the support they were offering would also be reciprocated if they ever needed it themselves. For instance, Sandy illustrates how group members would seek help from others if someone was unable to provide support, thus creating a ‘group dynamic’ which resulted in a greater overall sense of identification with the group:

*There was definitely a group dynamic*. *If…somebody in our village*…*couldn’t do a bit of shopping for somebody or pick up a prescription*, *because they had problems themselves*, *then they’d ask somebody else in the wider group*. *We’d all help out*. *Yes*, *we’re definitely part of a group*…*I think everybody mucked in* (Sandy)

Hayden stated that they felt a sense of community despite not having been helped themselves because they believed that they could call on support if they ever needed it, and there was an expectation that others would also feel the same

*’I think it is a whole community*. *And it is not about one group of people helping out another because it is an ever-changing landscape*. *We all recognise that if we are helping somebody today we might be asking for help tomorrow [] It is a whole* community’ (Hayden)

Rudy also describes how feeling a sense of mutual group support provided benefits for their own personal well-being, as it helped them get through the first lockdown:

*I kind of felt that that group is there*, *and I guess people are glad that you’re helping and so you feel like you’re part of something positive so it helps you manage the pandemic*. *It helps you to stop panicking about it a bit as well*, *I think*, *because like you feel like you’re doing something constructive and positive…in the face of this challenge… I guess being part of the group has helped me manage the pandemic for me as a person*. (Rudy)

Developing this point of collective community effort further, a shared identity either emerged or was reinforced through participants’ involvement in helping others. The pandemic provided opportunities for interactions to take place creating a shared space for a community identity to emerge that was based on mutual care and support. Supporting their own community was also highlighted in participants’ accounts of why they got involved in helping others. Following on from this sense of mutual support, when asked about their feelings towards their fellow volunteers and those that they helped, some participants reported that they had a more inclusive shared identity and did not necessarily feel that they were in separate psychological groups, thus rejecting the notion of ‘*us and them’* as illustrated by Ashley:

*You’ve got lots of activity and everybody weaving in and out of each other’s lives*. *There is no ’us and them’ of the people we help and the people who we don’t help*. *We had some volunteers who had to shield at one point and then came back and were able to help again*. *It’s all sort of a flux of interactions*, *really*, *rather than anything separate from each other*. (Ashley)

Max further illustrates this sense of mutuality and interconnectedness when elaborating further on their sense of connection between the helpers and those that were being helped:

*Int*: *Do you think that you feel part of one large group*? *Is it different smaller groups of the people that are helping and the people that are being helped, or is it just a bunch of individuals who are helping out other individuals?*

*Max*: *Definitely one big community*. *I wouldn’t say*, *it’s one group of people who help and one group of people who receive help*. *It has been very*, *I think*, *very fluid*. *Sometimes*, *somebody might be receiving help or needing help with something else*, *but then next week they come and they help with somethin*g *else*.

In summary then, this theme illustrates how participants’ involvement in helping others resulted in them feeling an enhanced sense of connection with their local community. Interestingly, they also reported an increased sense of mutual support, in that by helping others, they felt they could also draw upon such support if they needed it themselves, although only four out of 17 participants reported that they had been direct recipients of any support themselves. This suggests that the sense of mutual support derived more from feelings of psychological benefit, rather than relating to any tangible and/or instrumental offers of help from others. Finally, some participants also reported a more inclusive sense of shared community identity and rejected the notion that they were in a different psychological group to those that they were supporting. We shall now explore the second theme that focuses on whether such social connections endured throughout the period of this study.

### Enduring connections and barriers to continued participation

This second theme focuses on participants’ accounts of whether the social connections that emerged from their involvement in helping others endured, and what factors promoted or hindered such connections. The overall theme is organised into two more specific sub-themes; a) w*illingness for future engagement; and b) structural impediments to engagement*. In these sub-themes we explore the following issues: participants’ commitment to continue helping others; how their involvement was affected by the constraints in societal structures governing participants’ lives; and views of why local action was more conducive to their ongoing involvement compared to their engagement with wider-scale organisations which could discourage further activity.

#### Willingness for future engagement

As explored in the previous broad theme on shared identities, the sense of being part of a mutually supportive community influenced participants’ ongoing efforts to support others during the first UK lockdown. However, whether participants felt their involvement would continue into any future lockdowns was also an important issue to consider, especially given that this later became necessary to prevent infection spread during future waves of the pandemic. While ongoing involvement throughout the first lockdown varied among participants, all stated their intention that if it became necessary again, they would continue to be involved in helping others, and/or reactivating the support network that they had been involved in. For instance, Charlie highlights that while the mutual aid group that they were involved in had begun winding down by Summer 2020 due to a decrease in demand, it could quickly be re-started again if required:

Interviewer: *If we do have a second wave and local lockdowns*, *would you get involved again*?

Charlie: *Absolutely*. *Within a heartbeat*. *We’re not disbanding the group*. *What we’re doing is archiving it so it’s very easy to wake everybody up agai*n.

Many participants emphasised that the new social ties that had emerged through the creation of their local community groups were still active and important to them. Participants also commonly expressed a desire to maintain such connections beyond the first lockdown. For example, some expressed an intention to arrange community social events once the pandemic was over to allow this emergent community identity to be expressed in other ways, once it became possible to host face to face events safely (such as a street party), as described by Taylor:

*There has also been some talk of*, *when this is all over*, *let’s do something*. *Let’s have a street party…There is a sense that there is a latent community spirit that can’t quite be expressed right now but that people want to foster*. (Taylor)

As Taylor emphasises in the quote above, the sense of community that grew out of the support network during the pandemic was something they wanted to continue and grow further beyond the virtual interactions experienced during lockdown. This is particularly interesting, given Taylor’s initial lack of shared sense of identity that was reported previously. For some who initially began helping others remotely, seeing the members of these online groups in person further enhanced this sense of connection that was originally created online. Toni described the sense of community they experienced the first time they met face to face with other members of the online meditation group that had been created during the first lockdown:

*So*, *there’s a…park*…*and we met there with our group for the first time*…*and there was just a tremendous sense of community that I’ve not really experienced…just a sense of gratitude and a sense of togetherness* (Toni)

In addition to maintaining these social connections, some participants also described how they continued the cooperation that they had started during lockdown. These cooperative acts sometimes continued even though such help was no longer acutely needed, suggesting that the act itself had become a strategy to maintain a sense of social connection. For example, Stevie and their family maintained this sense of connection by continuing to look after a keyworker’s pet even after lockdown restrictions had been eased:

*We were walking a dog for someone who is a key worker*, *and we are still doing that now*. *They probably don’t really need us to walk their dog now*, *but we are still doing it*. (Stevie)

#### Structural impediments to continued engagement

While the UK saw a massive surge of volunteers offering to help others during the pandemic from March 2020 onwards, not all of those that came forward were used by official organisations [6]. This phenomenon was also reflected in our dataset and participants often reported that barriers to initially getting involved in supporting others were because of flaws in the wider-organisational structure. Participants emphasised issues with finding opportunities to get involved, such as a lack of official guidance regarding what to do and where to sign up. These issues led to people sometimes taking matters into their own hands to create a local support network as illustrated by Jesse:

*The local council*, *and I suppose the people*…*that were meant to be in charge…let us down*. *It took people*, *individuals*, *to do things*, *rather than the actual people who are meant to take charge* (Jesse)

With relation to those who had been involved in larger support networks and/or national organisations, even when there had been opportunities to help, the nature of how these larger groups operated made such engagement more difficult to manage for some participants. Hayden illustrates how not being able to meet requests for help within the large organisation that they had originally become involved with, were overwhelming volunteers and even causing some to leave the group:

*And the requests for help were being lost*. *Even within the reps group people were finding it completely overwhelming and were just leaving* (Hayden)

As emphasised above, the barriers to getting involved were mostly due to external factors (and not the participants’ lack of motivation to help others), such as decreased opportunities along with a lack of structure to advance from signing up to action. There was a similar framework for barriers to enduring involvement, with the structural factors decreasing or hindering ongoing engagement. When lockdowns were lifted and restrictions eased, the context changed, consequently affecting participants’ opportunities and possibilities to stay involved in support networks. For instance, Sydney described how a return to ‘normality’ arose from people regaining a sense of agency post lockdown leading to more doing their own shopping and other daily activities:

*people are just able to do their own thing*, *and things are a little bit more back to normal* (Sydney)

Sydney reported a decreased demand for support which affected some participants’ involvement in their local support groups. The return to ‘normality’ as the restrictions on staying at home were lifted also meant that the volunteers now faced other demands, such as returning to work. This return resulted in increased domestic demands for some, as most children in the UK were not able to attend school, and so needed to be home-schooled by their parents. For example, Ashley describes how child-care commitments hindered their ability to continue to support others once their partner had returned to work, and they became the primary carer for their children:

*My ability to help*, *if I’m the only person in the house and I’ve got the kids*, *obviously is very different than if my [partner] is around and one of us can nip out and do something for someone*. (Ashley)

As described above, the barriers to getting involved and staying involved in supporting others were related to factors beyond their own control such as decrease in demand, and the consequences of returning to ‘normal life’. In addition to these factors, participants also emphasised possible issues with large-scale organisations or wider support networks in relation to endurance of their involvement, preferring a localised structure for support during the pandemic. Furthermore, the local context in which the support occurred, enabled a shared community identity to both emerge and endure. This sense of connection with their immediate local community was differentiated from a broader community identity in various ways. For instance, Taylor emphasised the importance of the localized nature of the support group and its shared collective source. This was amongst other things, reflected in the name that they adopted to describe their group. The group was named after the road they lived in, as it related to a more localised identity rather than to a broader city-based identity. Having such a locally based identity made it easier for participants to get to know each other and identify more strongly with this local support group, thus creating more agency within the group and ownership of the support network:

*I think there is something important about it being named as such*, *that it felt very local*. *It wasn’t Brighton support group or Hove support group*. *This is our road* (Taylor)

As exemplified above, participants expressed the importance of being able to feel that it was their community. In their references to wider and/or national support schemes, participants did not refer to a sense of a shared community with these schemes, nor did they feel that their actions within them were effective. There was a unanimous preference for local groups and participants tended to perceive local groups as more effective than the wider national networks. This was explained through preferences for less bureaucracy and more direct communication in smaller, more localised support groups when compared to the more stratified structures of larger and/or national support networks. For instance, Corey contrasted the support group in their village with the support groups that were operating in the nearest sizable town, and emphasised their own explicit decision to avoid mirroring such a model:

*If you look at [XXXX] they’ve actually set up a massive organisation*. *It’s got its own finances*, *it’s got its own trustees and all that kind of stuff…[YYYY]…quite deliberately decided not to go in that direction*, *which is great because there’s a layer of bureaucracy we don’t have*. (Corey).

Along with the practical justification for less bureaucracy in favour of more localised community support, some participants emphasised that larger groups quickly became unwieldy—especially when the help that was needed was advertised via alerts on social media. The issue was explained in terms of the volume of notifications alerting people of requests for help and associated responses, which could quickly become overwhelming and result in users disengaging. This could render the use of social media to advertise requests for help counter-productive, as potential volunteers that disengaged from the platform would no longer receive requests for help:

*It was pretty useless actually*. *Because what happened at the beginning was that everybody was completely overloaded with notifications*…*So people were getting very*, *very stressed out*…*and they couldn’t keep up with the chat*. (Hayden)

In addition to becoming overwhelmed by the notifications, others talked about how the wider schemes felt very impersonal and/or inefficient. For example, some were explicitly critical of what they perceived as failings with the NHS volunteering scheme. Jules (who had previous clinical experience in the NHS) became involved in more localised mutual support groups because they did not feel that their involvement in the NHS volunteering scheme had been useful. Furthermore, they explained how they became increasingly frustrated because they felt that they were not utilised effectively due to the nature of how the requests for help came through, and that some volunteers were being wrongly contacted with offers of support when help was not actually needed:

*I’ve been called numerous times but never actually completed any of the tasks*. *I think because it’s done on your locality*, *because I live out in the countryside*, *I didn’t tend to get many calls*. *If I travelled through ZZZZ on my way to do some volunteering at the foodbank*, *I would suddenly get called all the time*…*It’s quite frustrating actually because a lot of the calls*, *when I’ve contacted the people they say*, *“Oh no*, *not another person calling me*.*” I’ve had to*, *on their behalf*, *try and take their name off the system because they keep getting calls when they haven’t actually requested it*. *It’s not worked very well* (Jules)

This theme has demonstrated how participants tended to keep their social connections active and unanimously expressed an interest in continuing to help others beyond the first acute phase of the pandemic. While some reported that their involvement in support groups had decreased over time due to lack of demand, none claimed that a lack of personal motivation was a factor in any discontinued volunteering. Some participants experienced external barriers to their involvement that were related to structural impediments, but most also acknowledged that as UK lockdown measures were progressively eased in the Summer of 2020, the need for help was no longer as acute and some previous social norms of behaviour that had existed before the lockdown, began to return. Furthermore, compared to wider national schemes, participants felt that the local community groups they became involved in created more efficient networks to provide ongoing support to others, whereas larger organisations were seen as less efficient. This was because localised groups encouraged closer interactions between helpers and helped, and they were also less impeded by the bureaucratic structures of larger organisations.

In summary, our findings section has highlighted how the pandemic created a space for opportunities to facilitate the emergence of a shared community identity, and how that identity facilitated continued engagement in support networks and continued social bonds within local communities. This shared identity was also often based on a sense of mutual support and was more inclusive than a simple dichotomy between ‘helper’ and ‘helped’. The preference for localised action and consequent sense of efficacy of action at this level highlight the importance of a shared local identity, and participants’ reduced sense of belonging to broader support schemes. Furthermore, the reasons for declining engagement in local support networks was often more related to external structural factors, rather than the participants’ own individual willingness, and they all intended to stay involved. However, due to decreased demands or changes in family structure (such as parents spending less time at home than they did during lockdown because of the return of work responsibilities), this engagement was no longer feasible. We will now discuss the theoretical and practical implications of our findings.

## Discussion

Participants provided rich accounts of their involvement in volunteering during the first COVID-19 lockdown in the UK from March-May 2020. They reported a strong sense of shared identity and community that either emerged from their own involvement or was further enhanced by any pre-existing social connections. They also expressed an overall preference for local support groups that was largely due to a sense that involvement in such groups was more effective than engaging in wider national support structures. Furthermore, participants reported that their desire to cooperate and their sense of shared identity endured throughout the first phase of lockdown. While involvement tailed off as the need for support in the community decreased, this was often due to external structural impediments (rather than lack of individual motivation or personal fatigue), and no one said they would not continue to help again if needed. Therefore, our findings address the original research topics of this study: 1) motivations for helping others and perceptions of such involvement; and 2) any potential barriers to enduring involvement. Given the rich dataset that we have found in support for these topics, we will now discuss the potential theoretical and practical implications of our study.

### Theoretical and practical implications

Our findings enhance theoretical and practical understanding of collective behaviour not only during the COVID-19 pandemic, but also of emergencies in general. Our study develops the notion of reciprocity in cooperation that has been found in previous research [9, 27] whereby people will not only help others during emergencies, but that they also expect to be helped in return (regardless of whether they personally receive any help or not), and this is due to the common identity that develops from a shared sense of adversity. This sense of mutual support, and more general enduring engagement through perceived ingroup involvement and support has also been found in other collective action contexts [45]. This also strengthens the notion that collective resilience and cooperation should be seen as emergent, dynamic, and reciprocal processes, rather than as inherent propensities that already exist in individuals and/or communities prior to major incidents occurring [23]. Therefore, our findings support and develop the theoretical assumptions of the Social Identity Model of Collective Psychosocial Resilience (SIMCPR), but also develop the notion that such collective resilience can endure during ongoing incidents (so long as the necessary broader structural support measures are in place). Therefore, ways of encouraging and fostering such behaviour should be considered in emergency planning and response guidelines, as has been suggested in previous practical recommendations to facilitate collective resilience in the public [8].

Participants’ preference for more localised identities in their support networks (relating to their own neighbourhood), supports previous work using social identity approaches that call for greater recognition of the shared identities that people can derive from their geographical locations [46] and of the psychological benefits that identification with one’s neighbourhood can bring [47]. Our study extends this previous work with the finding that such identities can emerge at even more localised levels (such as one’s street) and can be easier to maintain than broader superordinate identities (such as regional or national identities). Furthermore, members of such localised groups may find it easier to empathise with, and help other in-group members, as their sense of shared identity with others within their own locality may be stronger, thus creating a collective sense of togetherness with one’s geographical location. Therefore, ways of promoting and maintaining such localised identities to encourage increased community cooperation in future pandemics should also be considered.

Regarding practical implications for action during the COVID-19 pandemic, our findings on engagement with localised mutual aid groups could inform behavioural strategies to encourage compliance with specific lockdown restrictions. For instance, the current UK guidelines are to self-isolate for at least 5 days for those infectious with COVID-19 [48], and mass compliance with such regulations is needed to reduce transmission risk, but self-reported compliance with such guidelines can be low. However, recent evidence has shown that those who received social support (such as receiving help from neighbours to buy groceries) were more likely to adhere to self-isolation lockdown restrictions [49]. Furthermore, the Independent SAGE group of UK scientists has recommended that there should be more government support for those isolating [50], and perhaps such official support could also be extended to those involved in mutual aid to mitigate the structural constraints that can impede ongoing involvement. Hence, localised mutual aid schemes could encourage a more general sense of social support from one’s community that would facilitate greater compliance than wider and/or national efforts, as recipients of such support would have a greater sense of psychological connection with those providing it. This fits with stated priorities for psychological research during the pandemic [51] suggesting that making collective identities more salient (and hence encouraging greater concern for the well-being of others) could also encourage greater compliance with COVID lockdown restrictions. Furthermore, a sense of connection to the source of social support has been shown to influence cooperation during the pandemic, and that being either the recipient or provider of social support ‘*can mobilize prosocial actions in time of a collective threat’* [52, p.9].

There are also broader implications for policy and practice relating to emergency planning and response guidelines, as our findings support a growing evidence base that calls for greater recognition of the potential for public intervention in mass emergencies. Such intervention by ‘zero-responders’ was first recognised at an official level in the UK in the inquiry into the 2017 Manchester Arena bombing [53], but this focused primarily on acute one-off incidents, such as terrorist attacks. More recently, there has been a recognition within the UK government of the potential utility of spontaneous volunteers [15], but this has largely focused on how to incorporate them within existing support networks (such as local authorities and NGOs). While there is some evidence for local authority support for grass-roots communities in the UK [54], there is still comparatively less recognition of the potential support that such emergent networks can provide. Therefore, we would support calls for the increased democratisation of emergency planning procedures by facilitating more bottom-up approaches to mass emergency response—something that is vital during a global pandemic that requires mass behavioural change to prevent transmission spread.

### Limitations and future research

The primary limitation of our study is that it needs to be considered within the broader contextual factors in which the research happened. For instance, the interviews were conducted after the first phase of an unprecedented lockdown in the UK, and there would have been little with which participants could have compared their recent experiences. Furthermore, the funding scheme that made this research possible specified that the research had to relate to the Southeast of the UK, thus limiting the geographical area in which we were able to collect data. Also, as we wanted their involvement to still be fresh in participants’ minds, the interviews were conducted in summer 2020 (when restrictions were being temporarily eased in the UK), resulting in data being collected before future waves of the pandemic hit the UK from September 2020 onwards, necessitating further national lockdowns. Therefore, we cannot definitively conclude from this dataset whether participants followed through with their stated intentions to continue their engagement in mutual aid networks during subsequent lockdowns. This limitation is something future longitudinal research could consider—for example whether expressed intentions to act correlate with future behaviour.

Whilst we are confident that we have provided sufficient evidence to support the notion of shared community identity and collective resilience, we are also mindful that there is a possible risk of self-selection bias in our dataset, as we recruited people who were already involved in helping others. Hence, it could be argued that some of our participants were already predisposed to helping behaviour and may have already had a stronger sense of community than the general population. However, our participants also reported an increased sense of identity with their local community from their involvement above and beyond any residual community identity they may have already had. Therefore, any initial individual altruistic motives that participants may have already had, were amplified further by the collective identity that emerged from their involvement. Nevertheless, future research could employ wider-scale studies to investigate perceptions of involvement in mutual aid groups and explore the associations between different influencing factors.

A final potential limitation is that our participant sample had a clear gender imbalance, with fourteen of our seventeen participants identifying as female, and only three as male. However, this gender imbalance is in line with other evidence of helping during the pandemic [14] and with more general trends in volunteering [55]. Why there is such a gender imbalance in volunteering is open to speculation, but one possibility is that women in the UK are still disproportionally involved in caring and domestic responsibilities, and so they may be more socialised to help others than men. Nevertheless, more research to explore possible gender socialisation influences behind volunteering would be a welcome development in this field of study.

### Conclusion

Our study of involvement in helping others during the COVID-19 lockdown contributes to a growing body of evidence that supports more positive interpretations of human behaviour in emergencies than those that are often presented in popular discourse and/or outdated ‘mass panic’ models [8, 23]. Furthermore, such cooperation can be an enduring phenomenon, and while we do not claim that this support is un-ending, it seems that external structural issues are most likely to hinder continued involvement rather than any lack of personal motivation. Therefore, we suggest that there should be an increased focus on the potential for community resilience rather than vulnerability during the current pandemic. While we do not deny that there has been significant individual hardship, we suggest that it would be more effective to target support at those individuals that need it, rather than assuming there will be generalised collective vulnerability. Therefore, any lack of compliance with necessary COVID-19 restrictions, should not be seen as failings in public psychology and/or morality. Furthermore, rather than decrying human nature for any non-compliance, we should instead focus on how compliance can be enhanced through governmental support and by facilitating the ability of local communities to provide mutual aid and support to those that need it.

## Supporting information

S1 File The study interview schedule. (DOCX)
