# Supplementary material for: ‘All together now’: Facilitators and barriers to engagement in mutual aid during the first UK COVID-19 lockdown
Source: PLoS One. 2023 Apr 12;18(4):e0283080. doi: 10.1371/journal.pone.0283080 (PMC10096193; doi:10.1371/journal.pone.0283080)
Supplement: S1 File — (DOCX) [file pone.0283080.s001.docx]

**S1. The study interview schedule**

**Demographics**

- Age, Location, Gender, Employment status

**Intro questions**

- Where do you live? How long have you been living there for?
- Did you feel part of the local community before the pandemic?

**Initial motivation for involvement**

How did you **react** when you first heard about the pandemic?

How did you feel when you realised it was in your local area/region (eg SE of England)?

- What made you decide to help others? How did you find out about available support groups (mainstream media, social media, word of mouth etc.)
- What groups did you get involved in? Local neighborhood/street spontaneously set up in response to (COVID mutual aid groups), or more official pre-existing groups (Red Cross, faith-based groups, local govt etc)
- Did you register with the NHS volunteering scheme?
- If so, have you been called upon to help? What are your thoughts of NHS scheme?

**Effectiveness of involvement:**

How effective do you think your own actions to support others have been?

How effective do you think the group you have been involved with has been?

Are you still involved with this group and/or helping on your own initiative?

If a similar pandemic happened again, would you get involved?

**Shared identification and collective identity with those helped & others providing mutual aid**

Do you feel part of your local community more or less than before (or no change?)

How do you feel towards those who have been involved in helping others?

How do you feel towards those you have helped?

Overall, do you feel part of one large group, different smaller groups, or just lots of individuals helping out etc.

Is there one word or phrase you would use to describe the people who helped?

Have you felt supported by others during the pandemic? Who?

How do you think that people react in general when such incidents occur?

- - Do you think that people act competitively? Why/why not?
  - Do they express solidarity? Why/why not?

Are there any people/groups you feel negatively towards? If so, why?

**Change of views/actions throughout pandemic**

Has your view of the pandemic changed since you first heard about it? What kind of change?

Has your behaviour changed since you first heard about it? Why, what made it change?

Has anything about your involvement in helping surprised you?

Any surprises about the pandemic in general?
